# Impacts of socio-economic determinants, spatial distance and climate factors on the confirmed cases and deaths of COVID-19 in China

**DOI:** 10.1371/journal.pone.0255229

**Published:** 2021-07-27

**Authors:** Xiao-Dong Yang, Xin-Yi Su, Hong-Li Li, Ren-Feng Ma, Fang-Jie Qi, Yue-E Cao

**Affiliations:** 1 Department of Geography and Spatial Information Techniques/Center for Land and Marine Spatial Utilization and Governance Research, Ningbo University, Ningbo, China; 2 Ningbo Universities Collaborative Innovation Center for Land and Marine Spatial Utilization and Governance Research at Ningbo University, Ningbo, China; 3 Institute of East China Sea, Ningbo University, Ningbo, China; 4 Global Centre for Environmental Research, Advanced Technology Center (ATC) Building, Faculty of Science, The University of Newcastle, Callaghan, NSW, Australia; 5 Cooperative Research Centre for Contamination Assessment and Remediation of Environment (CRC CARE), The University of Newcastle, Callaghan, NSW, Australia; 6 School of Environmental and Geographical Science, Shanghai Normal University, Shanghai, China; 7 Institute of Resources and Environment Science, Xinjiang University, Urumqi, China; Institute of Geographic Sciences and Natural Resources Research (IGSNRR), Chinese Academy of Sciences (CAS), CHINA

## Abstract

This study is to assess the influences of climate, socio-economic determinants, and spatial distance on the confirmed cases and deaths in the raise phase of COVID-19 in China. The positive confirmed cases and deaths of COVID-19 over the population size of 100,000 over every 5 consecutive days (the CCOPSPTT and DOPSPTT for short, respectively) covered from 25th January to 29th February, 2020 in five city types (*i*.*e*., small-, medium-, large-, very large- and super large-sized cities), along with the data of climate, socio-economic determinants, spatial distance of the target city to Wuhan city (DW, for short), and spatial distance between the target city and their local province capital city (DLPC, for short) were collected from the official websites of China. Then the above-mentioned influencing factors on CCOPSPTT and DOPSPTT were analyzed separately in Hubei and other provinces. The results showed that CCOPSPTT and DOPSPTT were significantly different among five city types outside Hubei province (*p* < 0.05), but not obviously different in Hubei province (*p* > 0.05). The CCOPSPTT had significant correlation with socio-economic determinants (GDP and population), DW, climate and time after the outbreak of COVID-19 outside Hubei province (*p* < 0.05), while was only significantly related with GDP in Hubei province (*p* < 0.05). The DOPSPTT showed significant correlation with socio-economic determinants, DW, time and CCOPSPTT outside Hubei province (*p* < 0.05), while was significantly correlated with GDP and CCOPSPTT in Hubei province (*p* < 0.05). Compared with other factors, socio-economic determinants have the largest relative contribution to variance of CCOPSPTT in all studied cities (> 78%). The difference of DOPSPTT among cities was mainly affected by CCOPSPTT. Our results showed that influences of city types on the confirmed cases and death differed between Hubei and other provinces. Socio-economic determinants, especially GDP, have higher impact on the change of COVID-19 transmission compared with other factors.

## Introduction

The coronavirus disease 2019 (COVID-19) was first reported in Wuhan, the capital city of Hubei province, China in late December 2019 [1, 2]. COVID-19 is ascribed to the severe acute respiratory syndrome coronavirus 2 (SARS-CoV-2), a novel coronavirus [3]. Due to high contagiousness, COVID-19 quickly spread around the world [4, 5], the world health organization (WHO) has announced COVID-19 as a global public health emergency. Till 2^th^ September, 2020, a total of 258,117,1 90 confirmed cases and 856, 555 deaths had been reported around the world [6, 7].

The confirmed cases and deaths of COVID-19 may vary between city types (*i*.*e*., small-, medium-, large-, very large- and super large-sized cities) [8–12]. Some studies on droplet-mediated viral diseases like influenza showed that the proportion of pathogenic virus and the overall number of microorganisms in the air in large cities are significantly higher than that in small cities [13, 14]. The reason may be that higher population density and higher amount of interactions with other people in larger cities are conducive to the spread of virus [15, 16]. Affected by “Urban Turbidity Island Effect”, higher concentration of air contaminant is also conducive to virus transmission in larger cities [17, 18]. Because of big population base, the per capita medical and health resources in larger cities may be lower than that in small cities, leading to the increase of mortality [19]. As a result, large cities may have higher confirmed cases and deaths of COVID-19. On the contrary, some scholars have reported that better medical conditions and higher education level which are usually features of larger cities can reduce the transmission of droplet-mediated viral diseases [13, 15, 16, 20], while peoples in poorer and less educated smaller cities are more vulnerable to novel coronavirus [13, 21]. This indicates higher confirmed cases and deaths may be observed in smaller cities. Thus, whether the spread of COVID-19 is higher in larger cities or smaller cities, is still an issue under debate.

Confirmed cases and deaths of COVID-19 among different cities may be affected by socio-economic determinants [13, 22]. Virus infection is usually presented at a higher risk in large cities, because of enhanced virus spread caused by larger people mobility [12, 23]. Economically underdeveloped cities could invest little in prevention to the spread of virus, thus increasing the number of virus-infected people [13, 22, 24]. Additionally, climatic factors have also been confirmed to have an obvious impact on the change of the confirmed cases and deaths of COVID-19 [3, 25, 26]. For example, coronavirus is discovered to survive longer, and is more likely to spread in cooler cities [3, 27]. Influences of temperature, relative humidity, and precipitation on COVID-19 transmission all differ between coastal cities and inland cities [28, 29]. Spatial distance to the outbreak area of the virus also affects variations on the confirmed cases and deaths of COVID-19 among cities [30, 31]. Cities closer to the epicenter of the epidemic are usually at higher risk and their growth rate for virus infection and transmission are higher than that of peripheral cities [32, 33].

At present, however, most studies on the impacts of socio-economic determinants, climate factors and spatial distance on the confirmed cases and deaths of COVID-19 only investigated a single type of factors [15, 16, 19, 22, 29]. For example, Luo et al. reported that weather was related to the spread of COVID-19 in China [34]. Liu et al. studied the impact of meteorological factors on COVID-19 transmission in China [3]. Auler et al. and Prata et al. found that high temperature and intermediate relative humidity might favor the spread of COVID-19 in tropical region [25, 26]. Qiu et al. found that population posed a higher risk to virus transmission than other economic conditions [24]. Gross et al. reported that propagation of COVID-19 from Hubei to other provinces in China has an obvious relation with spatial distance in early time of the epidemic [35]. Hence, among these existing studies, influences of these above potential factors on COVID-19 transmission were not comprehensively analyzed. The relative importance of these contributors to the changes of the confirmed cases and deaths of COVID-19 remain unclear.

After large-scale outbreak of COVID-19 in Wuhan (the capital of Hubei province with 11 million residents), China’s government implemented many control measures in order to minimize the infection of virus to local residents [5, 36]. More specifically, a series of strict control measures, such as traffic control, home quarantine and medical aid from around the country, were carried out in Hubei province (especially the shut-down Wuhan) [36, 37]. But outside Hubei province, different control measures were taken, which mainly included: (1) shelter-in-place and quarantine for all confirmed cases and control traffic system to Hubei province on a national scale; (2) quarantining people from Wuhan or contacted to Wuhan people in the last one month and screening them by medical examination (e.g. CT Scan-Chest); and (3) limiting population migration nationwide [3]. These different control measures in Hubei and other provinces might have caused different relative contributions of socio-economic determinants, climate factors and spatial distance to the confirmed cases and deaths of COVID-19. However, limited studies have reported such difference at present [27].

In this study, in order to explore the relationship between COVID-19 transmission and city sizes, socio-economic determinants, climate factors as well as spatial distance, the confirmed cases and deaths of COVID-19 from 25^th^ January to 29^th^ February 2020, the data for above-mentioned four factors in all China’s cities were collected. Then relationships among them were analyzed using generalized linear mixed models (GLMMs) and hierarchical partitioning analysis (HPA). The aims of this study are to answer the following three questions: (1) Are there any differences in the confirmed cases and deaths of COVID-19 among different city types? (2) Are there any differences in relative contribution of city sizes, socio-economic determinants, climate and spatial distance to variability of the confirmed cases and deaths of COVID-19? and (3) Are these relative contributions different between Hubei province and other regions? Our results will provide a useful insight in explaining the variability of COVID-19 transmission (confirmed cases and deaths), the relevant influencing factors as well as the relative contribution of different factors. These results will give a practical guidance for drawing up the prevention and control measures of COVID-19 at present and in the future.

## Methods

### Ethics statement

No specific permissions were required for the described studies, because we did not carry out any experiments. All data used in this study are issued by China’s National Health Commission, China Meteorological Administration, Global Climate Dataset and Local City Weather Bureaus (see Table 1). All data is public and free to use.

**Table 1:** A summary of data resource.

Data type	Data sources	Website
The positive confirmed cases and deaths of COVID-19	China’s National Health Commission	http://www.nhc.gov.cn/xcs/yqtb/list_gzbd_3.shtml
	Provincial Health Commission of China	Web link can be found in China’s National Health Commission
Climatic factors (relative air humidity, average temperature and solar radiation intensity)	China Meteorological Administration	http://www.cma.gov.cn/
	Global Climate Dataset	http://www.worldclim.org/
Socio-economic determinants (GDP and population)	China’s National Statistical Yearbook	http://www.stats.gov.cn/tjsj/ndsj/
Spatial distance (DW and DLPC)	Baidu Map	https://map.baidu.com/@13531775.58,3466675.15,12z
City types	Soil Transaction Website of China	https://www.tuliu.com/read-81029.html

DW and DLPC are spatial distance of the target city to Wuhan city, and spatial distance between the target city and their local province capital city, respectively.

### Data collection

In this study, we chose 29^th^ January to 29^th^ February 2020 as our studied period because China National Health Commission (NHC) began to collect and release epidemic data across the whole country after 25th January [38]. It was also because that the period prior to March, 2020 is considered as the rise phase of COVID-19 epidemic in China [4, 5]. Chinese government’s control measures were most strictly enforced during this period. The daily officially reported positively confirmed case and deaths of COVID-19 were obtained from the official website of China’s National Health Commission, and the provincial health commission (including municipalities, and special administrative regions) (Table 1). In the meantime, climatic factors of all China’s cities were recorded from China Meteorological Administration and Global Climate Dataset (Table 1). In this study, we selected the relative air humidity, temperature and solar radiation intensity as representing climate factors. This is because these parameters has been reported as the most influencing meteorological factors on COVID-19 transmission in previous studies [3, 24, 26, 29].

Socio-economic determinants including GDP and population, for which the data at the end of 2018 were collected from the China’s National Statistical Yearbook because data for year 2019 and 2020 have not been released. GDP and population were chosen because they have decisive effects on factors that directly affected transmission and prevention of COVID-19, such as population mobility, education level and medical conditions [2, 13, 39]. Assuming that GDP and population would not change dramatically between two consecutive years, the impact of using the 2018 Statistical Yearbook data on our analysis would be negligible. Spatial distance included the distance of the target city to Wuhan city (DW for short), and distance between the target city and their local provincial capital city (DLPC, for short). DW and DLPC were calculated through Baidu maps, referring to the shortest traffic distances between cities (Table 1).

### Data analysis

The number of cities selected in this study is 334, which is far less than the total number of cities in China (approximately 661 in 2020). This is because the cities selected in this study are all cities where the conformed case of COVID-19 was reported from NHC, while the cities without the conformed case were not selected. All cities (324) were categorized into five types: small-, medium-, large-, very large- and super large-sized cities, as suggested from land transaction website of China (Table 1). The cities were mainly classified according to their resident population, GDP, cultural radiation area and urban built-up area (especially emphasizing on population). The Chinese State Council stated in 2014 that, cities with a population of more than 10 million, 5~10 million, 1~5 million, 0.5~1 million, and less than 0.5 million were defined as super large-sized, very large-, large-, medium- and small-sized cities, respectively. Previous studies have indicated that the epidemic intensity had trivial dependencies on population scale [2, 23]. In order to reduce this effect, the daily confirmed cases and deaths in population size of 100,000 were set as the initial data for data processing. Therefore, we used the positive confirmed cases and deaths over the population size of 100,000 at every 5 consecutive days (the CCOPSPTT and the DOPSPTT for short, respectively) as for the statistical analysis, with totally 8 time periods. In each time period, climatic factors are represented by their average of 5 consecutive days. Then, one-way ANOVA was used to compare the differences of CCOPSPTT and DOPSPTT among five city types at eight periods. Here we use eight periods to reduce the number of repetitive samples. Time was included as one analysis factor to investigate whether CCOPSPTT and DOPSPTT in different types of cities change with time. In one-way ANOVA, if the variance of the above indicators was homogeneous, the Tukey HSD was used to test the differences of CCOPSPTT and DOPSPTT among different cities as impacted by above mentioned influencing factors. Alternatively, if the variance was heterogeneous, Kruskal-Wallis test was used. The use and explanation of one-way ANOVA see Wilcox [40].

Based on the investigative data of 324 cities, GLMMs were used to test the multiple-influences of city sizes, socio-economic determinants, climate factors, spatial distance and time on CCOPSPTT and DOPSPTT of COVID-19. In GLMMs, city sizes were set as a random variable in order to reduce the dependencies of epidemic intensity on population scale. In addition, previous studies have suggested that variances of CCOPSPTT and DOPSPTT can be affected by time [1, 14]. After the outbreak of COVID-19, the spread of this virus from the outbreak area to other cities and the increase in infected persons within a city accumulated and shifted over time. Thus, in the course of data analysis, time was included. We set the starting day of data collection (25th January, 2019) as 0, and values of time were calculated as the interval of the other dates to this day. Hence, times of the eight studied periods were set as 0, 5, 10, 15, 20, 25, 30 and 35, respectively. Though the date of first officially confirmed case (29th January 2020) in Wuhan should be set to 0, our times were safe to use as they are mathematically logically equal with initially times of the first confirmed case. The only difference between the two referential times is a constant, which did not affect the statistical results. Moreover, impact of the confirmed cases on deaths cannot be ignored, because it is the most important direct factor causing death. Thus, we also set CCOPSPTT as an independent variable of DOPSPTT in GLMMs. The econometric models of CCOPSPTT and DOPSPTT are shown in (1)-(2).


$${CCOPSPTT}_{ct}=\alpha_{0}+\alpha_{1}C_{ct}+\alpha_{2}{SD}_{ct}+\alpha_{3}{SED}_{ct}+\alpha_{4}t+\varepsilon$$ (1)

$${DOPSPTT}_{ct}=\alpha_{0}+{\alpha_{1}{CCOPSPTT}_{ct}+\alpha }_{2}C_{ct}+\alpha_{3}{SD}_{ct}+\alpha_{4}{SED}_{ct}+\alpha_{5}t+\varepsilon$$ (2)

In the models, *C*, *SD* and *SED* represent climate factors, spatial distance and socio-economic determinants, respectively. Their sets are respectively expressed in Formula 3–5. *c* and *t* represent city size and time respectively. *SRI*, *AT* and *RAH* are solar radiation intensity, average temperature and relative air humidity, respectively. *ε* is the random error; which is formed by the random action of many factors in the process of measurement and statistics. If the significance of regression coefficients, such as *α* and *β*, is less than 0.05, suggesting that they have the significantly impacts on CCOPSPTT or DOPSPTT.


$$C_{ct}=\beta_{1}{SRI}_{ct}+\beta_{2}{AT}_{ct}+\beta_{3}{RAH}_{ct}$$ (3)

$${SD}_{ct}=\gamma_{1}{DLPC}_{ct}+\gamma_{2}{DW}_{ct}$$ (4)

$${SED}_{ct}=\theta_{1}{GDP}_{ct}+\theta_{2}{Population}_{ct}$$ (5)

Finally, we used HPA to compare the relative contributions of these factors on CCOPSPTT and DOPSPTT. Here, the relative contribution was the proportion of each independent variable from the goodness-of-fit measures across all variable combinations in a hierarchy, which indicated weight of one variable versus other independent variables that obviously contributed to COVID-19 transmission. When performing GLMMs and HPA, all data were standardized by logarithmic transformation in order to avoid the trivial influence of the non-normal data on statistical results (*e*.*g*., much data of CCOPSPTT and DOPSPTT in different city types did not conformed to the normal distribution, see Tables 2 and 3; *SD* > Mean indicates non-normal distribution). As COVID-19 control conditions were much more complicated in Hubei compared with other provinces, like lockdown and mass screening, data analysis of Hubei and other province were performed separately. The use and explanation of GLMMs and HPA see Dichmont [41], Lai and Peres-neto [42]. In the data analysis processes of GLMMs and HPA, socio-economic determinants, climate factors, spatial distance and time were independent variables, while CCOPSPTT and DOPSPTT were dependent variables. Remarkably, we had not defined one or several specific variables as control variables, and then used the econometric model to analyze relationships of explanatory variables with CCOPSPTT and DOPSPTT. Instead, we defined all independent variables as explanatory variables. In this case, seven independent variables, including solar radiation intensity, average temperature, relative air humidity, time, GDP, population, DW and DLDP, have the an equivalent logical impacts on CCOPSPTT and DOPSPTT. They were all explanatory variables, not control variables.

**Table 2:** Differences in the confirmed cases over population size per 100000 at every 5 consecutive days (CCOPSPTT) among five types of cities outside and within Hubei province from 25th January to 29th February, 2020.

Types	Date	City types					Statistical result		
		SL	VL	L	M	S	chi-squared	*df*	*p*-values
Hubei province	25^th^ Jan	6×E-3	/	(3±2)×E-4a	(5±4)×E-4a	(1±2)×E-4a	3.66	2	0.16
	30^th^ Jan	0.02	/	(3±1)×E-3a	(2±1)×E-3a	(10±8)×E-4a	3.67	2	0.16
	4^th^ Feb	0.06	/	(5±2)×E-3a	(4±4)×E-3a	(2±1)×E-3	5.94	2	0.10
	9^th^ Feb	0.09	/	(5±3)×E-3a	(2±2)×E-3a	(2±1)×E-3a	2.62	2	0.27
	14^th^ Feb	0.21	/	(3±2)×E-3a	(2±2)×E-3a	(2±1)×E-3a	1.02	2	0.60
	19^th^ Feb	0.07	/	(3±4)×E-3a	(4±4)×E-3a	(2±3)×E-3a	0.13	2	0.94
	24^th^ Feb	0.02	/	(7±5)×E-4a	(4±2)×E-4a	(3±4)×E-4a	1.50	2	0.47
	29^th^ Feb	0.02	/	(1±1)×E-4a	(2±2)×E-5a	(2±2)×E-5a	0.99	2	0.61
Other provinces	25^th^ Jan	(2±2)×E-4a	(4±4)×E-5b	(2±3)×E-5c	(2±3)×E-5c	(0.6±1)×E-5d	105.38	4	**<0.001**
	30^th^ Jan	(5±4)×E-4a	(2±3)×E-4a	(8±8)×E-5b	(7±7)×E-5b	(3±4)×E-5c	123.61	4	**<0.001**
	4^th^ Feb	(7±5)×E-4a	(3±3)×E-4a	(1±1)×E-4b	(1±1)×E-4b	(4±6)×E-5c	111.96	4	**<0.001**
	9^th^ Feb	(4±3)×E-4a	(2±2)×E-4a	(0.9±1)×E-4b	(0.7±1)×E-4b	(3±6)×E-5c	99.29	4	**<0.001**
	14^th^ Feb	(2±2)×E-4a	(1±1)×E-4a	(6±7)×E-5b	(4±6)×E-5b	(2±4)×E-5c	82.88	4	**<0.001**
	19^th^ Feb	(7±6)×E-5a	(4±5)×E-5a	(1±2)×E-5bc	(1±2)×E-5b	(0.8±2)×E-5c	57.18	4	**<0.001**
	24^th^ Feb	(3±4)×E-5a	(0.6±2)×E-5b	(0.4±3)×E-4b	(2±7)×E-6bc	(2±7)×E-6c	50.88	4	**<0.001**
	29^th^ Feb	(0.9±3)×E-5a	(1±6)×E-5ab	(0.9±3)×E-6ab	(0.8±8)×E-6b	(0.5±2)×E-5ab	11.88	4	**<0.05**

Different lowercase letters indicate the significant differences in the confirmed cases among the different cites, whereas the same uppercase letters show the non-significant differences. SL, VL, L, M and S refers to super large-, very large-, large-, medium- and small-sized cities, respectively. “/” indicates no data in VL because there is no very large-sized city in Hubei province.

**Table 3:** Differences in deaths over population size per 100000 at every 5 consecutive days (DOPSPTT) among five types of cities outside and in Hubei province from 25th January to 29th February, 2020.

Types	Date	City types					Statistical result		
		SL	VL	L	M	S	chi-squared	*df*	*p*-values
Hubei province	25^th^ Jan	5×E-4	/	(8±8)×E-6a	(6±8)×E-6a	(1±3)×E-6a	1.95	2	0.38
	30^th^ Jan	1×E-3	/	(3±4)×E-5a	(2±4)×E-5a	(3±2)×E-5a	1.26	2	0.53
	4^th^ Feb	2×E-3	/	(5±3)×E-5a	(3±5)×E-5a	(5±5)×E-5a	1.78	2	0.41
	9^th^ Feb	3×E-3	/	(8±4)×E-5a	(5±8)×E-5a	(2±2)×E-5a	4.64	2	0.10
	14^th^ Feb	4×E-3	/	(20±9)×E-5a	(9±8)×E-5a	(6±4)×E-5a	2.81	2	0.25
	19^th^ Feb	5×E-3	/	(10±8)×E-5a	(8±7)×E-5a	(4±3)×E-5a	3.88	2	0.14
	24^th^ Feb	5×E-3	/	(8±8)×E-5a	(5±7)×E-5a	(2±3)×E-5a	2.51	2	0.29
	29^th^ Feb	2×E-3	/	(5±2)×E-5a	(3±3)×E-5a	(2±2)×E-5a	3.36	2	0.19
Other provinces	25^th^ Jan	(0.6±2)×E-6a	0.00±0.00a	(0.2±1)×E-6a	0.00±0.00a	0.00±0.00a	9.28	4	0.05
	30^th^ Jan	(2±4)×E-6a	0.00±0.00b	(0.6±4)×E-6b	0.00±0.00b	0.00±0.00b	44.56	4	**<0.001**
	4^th^ Feb	(1±5)×E-6a	0.00±0.00b	(0.3±2)×E-6b	(0.6±3)×E-6b	0.00±0.00b	5.74	4	**<0.05**
	9^th^ Feb	(2±4)×E-6a	(1±5)×E-6ab	(1±4)×E-6b	(0.5±2)×E-6b	(0.3±2)×E-6b	9.78	4	**<0.05**
	14^th^ Feb	(0.6±1)×E-5a	(0.7±2)×E-6b	(0.8±4)×E-6b	(1±8)×E-6b	(0.3±2)×E-6b	21.85	4	**<0.001**
	19^th^ Feb	(4±8)×E-6a	(0.7±2)×E-6ab	(0.5±2)×E-6b	(0.9±7)×E-6b	(0.2±1)×E-6b	16.42	4	**<0.01**
	24^th^ Feb	(2±5)×E-6a	(0.3±2)×E-6ab	(0.6±2)×E-6ab	(0.1±1)×E-6b	(0.8±9)×E-7b	16.02	4	**<0.01**
	29^th^ Feb	(2±9)×E-6a	(0.3±2)×E-6a	(0.2±1)×E-6a	0.00±0.00a	(0.2±2)×E-6a	5.01	4	0.29

Different lowercase letters indicate the significant differences in deaths among the different cites, whereas the same uppercase letters show the non-significant differences. The introduction of SL, VL, L, M, S and “/” see Table 2.

The creditability of GLMMs and HPA results was determined by determinate coefficient (*R*^2^) and *p*-values. High *R*^2^ and *p*-values <0.05 indicated that statistical results were credible. Besides, we also tested the robustness of the model to ensure the credibility of the research results. In this study, since independent variables, especially climate factors, are highly correlated between adjacent time observation points, their values at time *t* not only have an impact on CCOPSPTT and DOPSPTT, but also have a significant impact at time *t*+1. Therefore, our model is time-delayed. According to the results of Jiang et al [43], Xun and Halbert [44], the robustness of the time-delay model can be tested with the data of one lag period. More specifically, the dependent variables can be estimated by the independent variables with a lag of one period (*i*.*e*., lag regression or time-delay model). When the regression parameters, like *p*-value, *R*^2^ and estimate coefficients, of time-delay and baseline (original) models are close to each other, it indicates that our model has high robustness. All data processing was conducted in R. 3.4.3 software.

## Results

### Differences in the CCOPSPTT and DOPSPTT of COVID-19 among five city types

Outside Hubei province, there was a significant difference on CCOPSPTT among the five city types from 25^th^ January to 29^th^ February, 2020 (*p* < 0.05) (Table 2). In most cases, CCOPSPTT decreased in the order of super large- ≥ very large- > large- ≥ medium- > small-sized cities (Table 2). Within Hubei province, CCOPSPTT in the only super large-sized city (Wuhan) were obviously higher than other cities. Additionally, the gap of CCOPSPTT between Wuhan and other cities in Hubei province continuously increased with time (Table 2). For example, on 25^th^ January, 2020, CCOPSPTT in Wuhan was 6×E-3, which was significantly higher than that of large-sized cities (3×E-4), medium-sized cities (5×E-4) and small-sized cities (1×E-4). Wuhan was more than 12 times higher than other cities on CCOPSPTT (Table 2). On 29^th^ February, 2020, the CCOPSPTT in Wuhan, large-, medium- and small-sized cities of Hubei province were 0.02, 1×E-4 (average), 2×E-5 (average) and 2×E-5 (average), respectively. There was over 200 times higher CCOPSPTT in Wuhan and in other cities (Table 2). In all sampling dates, difference of CCOPSPTT among large-, medium- and small-sized types of cities in Hubei was not significant (*p* > 0.05) (Table 2).

DOPSPTT showed no significant difference among five city types outside Hubei province on 25^th^ January and 29^th^ February, 2020 (*p* > 0.05) (Table 3). But during the six sampling periods in between these two dates outside Hubei province, DOPSPTT in super large-sized cities were significantly higher than that in other cities (*p* < 0.05). DOPSPTT showed no significant difference among other four (very large-, large-, medium- and small-sized cities) types of cities outside Hubei province (*p* > 0.05) (Table 3). In Hubei province, DOPSPTT of the only super large-sized city (Wuhan) were obviously higher than that of other cities at all sampling dates. The gap of DOPSPTT between Wuhan and other Hubei cities also continuously increased with time. The DOPSPTT also showed no significant difference among large-, medium- and small-sized cities (*p* > 0.05) (Table 3).

### Multiple relationships of socio-economic determinants, climate, spatial distance and time to the CCOPSPTT and DOPSPTT of COVID-19

The GLMMs results showed high *R*^2^ values (> 0.10) and significant *p*-values (< 0.001) in all analysis (Tables 4 and 5). Our results also showed that the time-delay and the baseline models have similar regression parameters. For example, in the GLMMs of COPSPTT, the *p*-values of population, GDP, DW, relative air humidity, solar radiation intensity, average temperature and time of the two models, were all less than 0.05 outside Hubei provinces. The positive or negative forms of regression coefficients of all independent variables in the two models were consistent (Table 4). *R*^2^ difference ranged from 0.01 to 0.04, and there was no obviously between the two models. These suggested that our model has high robustness (Tables 4 and 5). The variability of CCOPSPTT and DOPSPTT among five city types could be affected by socio-economic determinants, climate, spatial distance and time.

**Table 4:** Results of GLMs in relationship of the confirmed cases over population size per 100000 at every 5 consecutive days (CCOPSPTT) with socio-economic determinants (GDP and population), spatial distance (DW and DLPC), climate factors (relative air humidity, solar radiation intensity and average temperature) and time in cities outside and within Hubei province.

Types	Regression parameters	Baseline model			Time-delay model		
		Coefficients			Coefficients		
		*Estimate*	*SE*	*p-value*	*Estimate*	*SE*	*p-value*
Hubei province	Intercept	-0.02	0.01	0.28	-0.02	0.01	0.31
	Population (Ten thousand)	-1×E-5	9×E-6	0.20	-1×E-5	9×E-6	0.23
	GDP (Hundreds of millions RMB)	5×E-6	6×E-7	<0.001***	4×E-6	5×E-7	<0.001***
	DW (km)	-1×E-5	1×E-5	0.25	-1×E-5	1×E-5	0.23
	Relative air humidity (%)	2×E-4	2×E-4	0.11	1×E-4	2×E-4	0.12
	Solar radiation intensity (h)	5×E-4	4×E-4	0.27	4×E-4	4×E-4	0.26
	Average temperature (°C)	-1×E-3	6×E-4	0.06	-1×E-3	6×E-4	0.07
	Time (d)	4×E-4	3×E-4	0.11	3×E-4	3×E-4	0.12
	*AIC*	-635.68			-643.23		
	Marginal *R*^*2*^	0.52			0.51		
	Conditional *R*^*2*^	0.52			0.51		
Other provinces	Intercept	5×E-5	9×E-6	<0.001***	4×E-5	9×E-6	<0.001***
	Population (Ten thousand)	9×E-8	1×E-8	<0.001***	8×E-8	1×E-8	<0.001***
	GDP (Hundreds of millions RMB)	1×E-8	9×E-10	<0.001***	1×E-8	1×E-9	<0.001***
	DW (km)	-2×E-8	6×E-9	<0.01**	-2×E-8	6×E-9	<0.01**
	DLPC (km)	-6×E-9	2×E-8	0.75	-5×E-9	2×E-8	0.75
	Relative air humidity (%)	-4×E-8	3×E-8	<0.05*	-4×E-8	3×E-8	<0.05*
	Solar radiation intensity (h)	3×E-6	8×E-7	<0.001***	4×E-6	7×E-7	<0.001***
	Average temperature (°C)	4×E-7	3×E-7	<0.05*	3×E-7	3×E-7	<0.05*
	Time (d)	-4×E-6	3×E-7	<0.001***	-5×E-6	4×E-7	<0.001***
	*AIC*	-32617.20			-32634.55		
	Marginal *R*^*2*^	0.33			0.32		
	Conditional *R*^*2*^	0.33			0.32		

The introduction of DW and DLPC see Table 1. The robustness of the model was tested by the similarity of regression coefficients between the baseline and time-delay models. If the regression coefficients are close to each other between the two models, indicating that our model has high robustness.

**Table 5:** Results of GLMs in relationship of deaths over population size per 100000 at every 5 consecutive days (DOPSPTT) with the CCOPSPTT, socio-economic determinants, spatial distance, climate factors and time in cities outside and within Hubei province.

Types	Regression parameters	Baseline model			Time-delay model		
		Coefficients			Coefficients		
		*Estimate*	*SE*	*p-value*	*Estimate*	*SE*	*p-value*
Hubei province	Intercept	-3×E-4	3×E-4	0.43	-4×E-4	3×E-4	0.41
	Population (Ten thousand)	-1×E-7	2×E-7	0.67	-2×E-7	2×E-7	0.62
	GDP (Hundreds of millions RMB)	1×E-7	2×E-8	<0.001***	1×E-7	2×E-8	<0.001***
	DW (km)	-4×E-7	2×E-7	0.24	-3×E-7	2×E-7	0.29
	Relative air humidity (%)	2×E-6	3×E-6	0.63	1×E-6	3×E-6	0.57
	Solar radiation intensity (h)	-2×E-6	9×E-6	0.85	-2×E-6	9×E-6	0.82
	Average temperature (°C)	-1×E-5	1×E-5	0.41	-1×E-5	1×E-5	0.36
	Time (d)	9×E-6	6×E-6	0.09	1×E-7	6×E-6	0.06
	The CCOPSPTT	0.01	2×E-3	<0.001***	0.01	2×E-3	<0.001***
	*AIC*	-1553.32			-1569.39		
	Marginal *R*^*2*^	0.82			0.79		
	Conditional *R*^*2*^	0.84			0.80		
Other provinces	Intercept	-9×E-7	3×E-7	<0.001***	-9×E-7	3×E-7	<0.001***
	Population (Ten thousand)	1×E-9	3×E-10	<0.001***	9×E-8	3×E-10	<0.001***
	GDP (Hundreds of millions RMB)	6×E-11	2×E-11	<0.05*	5×E-11	2×E-11	<0.05*
	DW (km)	3×E-10	2×E-10	<0.05*	3×E-10	2×E-10	<0.05*
	DLPC (km)	5×E-11	5×E-10	0.92	6×E-11	6×E-10	0.92
	Relative air humidity (%)	1×E-9	9×E-10	0.22	1×E-9	1×E-11	0.21
	Solar radiation intensity (h)	-1×E-8	2×E-8	0.63	-1×E-8	2×E-8	0.64
	Average temperature (°C)	-9×E-9	9×E-9	0.31	-9×E-9	9×E-9	0.34
	Time (d)	2×E-8	8×E-9	<0.05*	1×E-8	8×E-9	<0.05*
	The CCOPSPTT	4×E-3	6×E-4	<0.001***	3×E-3	6×E-4	<0.001***
	*AIC*	-48088.99			-48101.99		
	Marginal *R*^*2*^	0.14			0.11		
	Conditional *R*^*2*^	0.14			0.10		

Instruction of the CCOPSPTT, social economy, climate factors, DW, DLPC and the robustness test as suggested in Tables 1 and 4.

Outside Hubei province, GDP (positive), population (positive), DW (negative), relative air humidity (negative), solar radiation intensity (positive), average temperature (positive) and time (positive) had a significant regression relationship with CCOPSPTT (*p* < 0.05). In contrast, DLPC had no significant correlation (*p*> 0.05) with CCOPSPTT (Table 4). In Hubei province, only GDP (positive) had a significant regression relationship with CCOPSPTT (*p* < 0.05), whereas other factors did not (*p* > 0.05) (Table 4).

Regarding DOPSPTT, GDP, population, DW, time and CCOPSPTT showed the significant positive influences on it outside Hubei province (*p* < 0.01) (Table 5). However, in Hubei province, the variability of DOPSPTT was significantly affected (positive) by CCOPSPTT and GDP (*p* < 0.05), but not related to other factors (*p* > 0.05) (Table 5).

**Fig 1:**
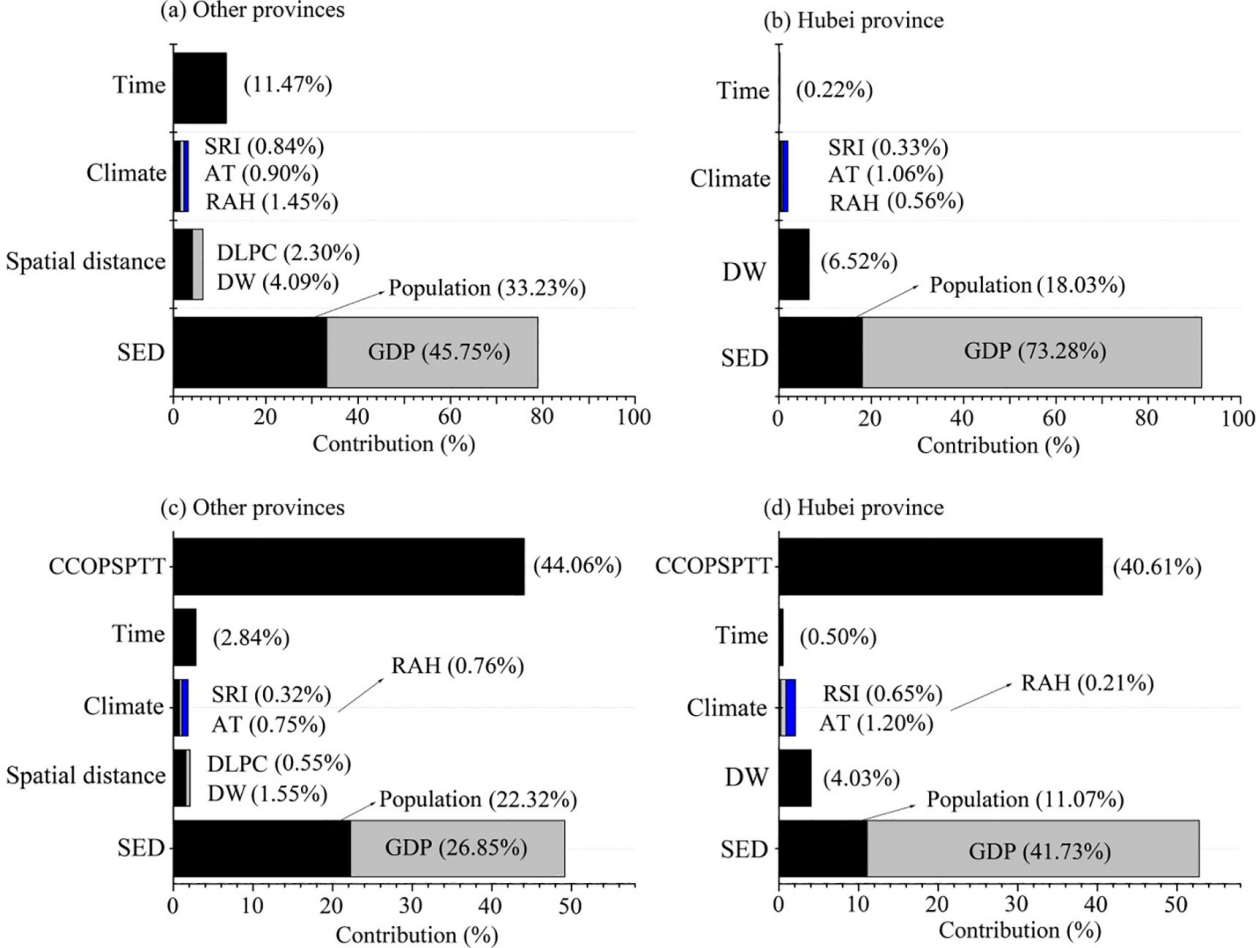
The relative contributions of different factors to the confirmed cases (a and b) and deaths (c and d) over population size per 100000 at every 5 consecutive days (CCOPSPTT and DOPSPTT). Instruction of DW and DLPC as suggested in Table 3. SED is socio-economic determinants. SRI, AT and PAH is solar radiation intensity, average temperature and relative air humidity, respectively.

The HPA results showed high *R*^2^ values (*R*^2^ of CCOPSPTT and DOPSPTT in Hubei and other provinces were 0.45, 0.30, 0.82 and 0.49, respectively) and significant *p*-values (< 0.001) in all analysis, indicating that HPA can be good used to decompose the relative contribution of each influencing factor to CCOPSPTT and DOPSPTT. This also suggested our results have high credibility (Fig 1). Among cities outside Hubei province, the results of HPA showed that the relative contributions of socio-economic determinants, climate, spatial distance and time to the change of CCOPSPTT were 78.90%, 3.20%, 6.40% and 11.50%, respectively. Specifically, the relative contribution of GDP (45.72%) was obviously higher than that of population (33.23%). DW (4.09%) contributed significantly higher than DLPC (2.30%) on CCOPSPTT (Fig 1A). All climatic factors had relatively lower relative contributions to CCOPSPTT (relative air humidity 1.45%; average temperature 0.90%; and solar radiation intensity 0.84%) (Fig 1A). In Hubei province, the relative contributions of different factors to variability of CCOPSPTT among cities were ordered as socio-economic determinants (91.31%) > spatial distance (6.52%) > climate (1.95%) > time (0.22%) (Fig 1B). The relative contribution of GDP (73.28%) was significantly higher than that of population (18.03%), while average temperature (1.06%) contributed slightly higher to that of relative air humidity (0.56%) and solar radiation intensity (0.33%) (Fig 1B). The relative contributions of GDP, spatial distance and average temperature to CCOPSPTT were obviously higher in Hubei province than those of other provinces (Fig 1A and 1C).

The relative contributions to DOPSPTT were higher from CCOPSPTT (44.06%) and socio-economic determinants (44.06%) than from spatial distance (2.10%), climate (1.83%) and time (2.84%) in cities outside Hubei province (Fig 1C). Particularly, the contribution of GDP (26.85%) was higher than that of population (22.32%). Contribution of DW (1.55%) was more than that of DLPC (0.55%), while the relative contributions of all climate factors were less than 0.76% (Fig 1C). In Hubei province, the relative contributions of CCOPSPTT, socio-economic determinants, climate, DW and time to DOPSPTT were 40.61%, 52.80%, 2.06%, 4.03% and 0.50%, respectively (Fig 1D). Among the sub-factors, the relative contribution from GDP (41.73%) was obviously higher than that of population (11.03%), and the relative contributions of all climatic factors were less than 1.20%. Meanwhile, GDP, spatial distance and average temperature showed higher contributions to DOPSPTT in Hubei than that in other provinces, while CCOPSPTT exhibited the opposite trend (Fig 1B and 1D).

## Discussion

### Relationship between city types and the confirmed cases and deaths of COVID-19

The difference of CCOPSPTT among five city types outside Hubei province (Table 2) suggested that city size and developmental level linked with the infection and transmission of COVID-19. The population and economic activities of super large- and very large-sized cities are significantly higher than those of other types of cities [9, 40]. In addition, due to the developed traffic system, population mobility in super large- and very large-sized cities are higher than those in large-, medium- and small-sized cities [32, 41]. These make population density and the amount of interactions with other people in super large- and very large-sized cities are higher than those of other cities [45]. According to the theory of infectious disease, the increases of population density and the amount of interactions with other people will improve the transmission risk of the epidemic [2, 23, 39, 46]. As a result, novel coronavirus would spread faster in super large- and very large-sized cities, resulting in the large number of CCOPSPTT. In addition, as suggested from Zhu et al. [17], Wu et al. [18] and Heibati et al. [28], urban climate factors and pollution status also affected the spread of novel coronavirus. The increases of contaminant concentration and air humidity in cold season both are beneficial to the spread of novel coronavirus [29]. Under the same geographical conditions, such as the location from the ocean and altitude, the pollutant concentration and air humidity in larger-sized cities are significantly higher than those in small- and medium-sized cities due to the influences of urban “Heat Island Effect” and “Turbidity Island Effect” [17, 18, 47]. Therefore, differences in climate factors and pollution status can also cause differences in the spread of novel coronavirus among cities with different sizes.

Among five city types, CCOPSPTT showed no significant difference between super large- and very large-sized cities, as well as between large- and medium -sized cities (Table 2). This may be determined by the complex relationships among population, population mobility, and the degree of the implementation of control measures [15, 16, 24]. For example, although super large-sized cities have larger population and higher population mobility, where the implementation of epidemic control measures would be more difficult compared with very large-sized cities. Beside, at present, people’s cognition on the role of income (GDP or GDPP) on COVID-19 transmission would be controversial because at an aggregate level higher degrees of economic development (*i*.*e*., a higher GDP) might promote higher population mobility, *e*.*g*., potentially favoring the spread of epidemics. But at the same time, a richer region should have larger amount of resources to put into place more effective control interventions. On the other hand, during a sudden and rapid period of COVID-19 outbreak, a larger GDP implying a potentially more educated population; which might allow the adoption of individual protective measures at a higher rate. This suggests that the potential role of GDP on COVID-19 can be complicated between different city types. This would also explain the insignificant difference of CCOPSPTT between super large- and very large-sized cities, as well as between large- and medium-sized cities.

Except for Wuhan, CCOPSPTT among large-, medium- and small-sized cities in Hubei province was not significantly different (Table 2). This indicated the change of confirmed cases from large- to small-sized cities was affected little by the city size and development level; which showed the opposite trend with cities outside Hubei province that significantly differed (Table 2). The difference in change of CCOPSPTT between Hubei and other province with city sizes may be related to China’s extreme control measures, such as strict traffic control and home quarantine [33, 36]. Wuhan was shut down on 23^th^ January, while traffic controls in other cities of Hubei province started after 28^th^ January, 2020. Home quarantine and traffic control could block the transmission of coronavirus [36]. Thus, change in the confirmed cases would be the effects of population mobility before the closure of Wuhan. As Wuhan is the capital city serving as the economic and cultural center, many virus carriers would have moved from Wuhan to smaller cities surrounded before the traffic control started. Within Hubei province, there might be no significant difference in the number of population mobility from Wuhan to surrounding small cities due to the developed transportation and the large population base. After traffic control and home quarantine, the increase in confirmed cases might be due to intra-family infection [37, 48]. Therefore, similar population mobility and similar extent of intra-family infection caused an identical CCOPSPTT value between large-, medium- and small-sized cities in Hubei province (Table 2). However, outside Hubei province, the number of people from Hubei province was higher in larger cities than that in small and medium-sized cities, because the former have closer contact with Hubei province’s cities, especially Wuhan, in terms of personnel flow and business exchanges [5, 32]. Such difference made CCOPSPTT of larger cities higher than that of small and medium-sized cities.

The DOPSPTT in super large-sized cities were higher than that in other cities, whereas there was no significant difference between very large-, large-, medium- and small-sized cities both in Hubei and other provinces (Table 3). This may be explained by the complicated influence from many factors, such as the confirmed cases, medical conditions, Intensive care unit (ICU) per capita and China’s control measures [5, 33, 36]. Although super large-sized cities have better medical conditions, but a higher confirmed cases and lower ICU per capita may lead to higher rate of deaths than that of other small cities. China’s strong control measures, such as traffic control, home quarantine, free medical treatment, testing and isolation of asymptomatic cases, has made the vast majority of COVID-19 patients and carriers treated and controlled [33, 37]. Thus, DOPSPTT had not differed between cities with different sizes. In Hubei province, in addition to the above several control measures, a large number of doctors and medical supplies were provided to these cities, and a large number of mobile cabin hospital were constructed [19, 32, 49]. Local hospitals have sufficient medical resources and equipment to treat patients. Therefore, DOPSPTT were not significantly different between very large-, large-, medium- and small-sized cities (Table 3). As an exception, CCOPSPTT and DOPSPTT of COVID-19 in Wuhan were obviously higher than in other cities of Hubei province (Tables 2 and 3). This is because Wuhan was the only large-scale epidemic area in China within our data survey period (from 25^th^ January to 29^th^ February). Government disease controls and the spread patterns of COVID-19 were different between Wuhan and other cities [4, 20]. Specific explanations can be summarized in the following aspects: (1) the difference between the proportion of the elderly with novel coronavirus in the total population in Wuhan and other cities. The population with high susceptibility and mortality of novel coronavirus was mainly elderly people [4, 19]. Wuhan was the first city to report COVID-19. The virus has spread widely in Wuhan as local authorities try to contain all aspects of the epidemic, leading to a higher proportion of the elderly infected with the virus. But outside Wuhan, the early detection and isolation of the virus effectively controlled the spread of the epidemic among the elderly; (2) the influences of timely medical treatment and epidemic prevention on the spread of novel coronavirus [19, 49]. Shutdown of Wuhan helped other cities to win a valuable time for epidemic prevention and control, thereby reducing the number of infections and deaths. At the same time, the implementation of effective measures avoided a run on medical resources, leading to a relatively low fatality rate [5, 36, 37]. However, in the early stage of the outbreak of the epidemic in Wuhan, many patients may not receive timely treatment due to a sharp increase in the number of infected person and the shortage of medical resources, leading to a high mortality rate [19]; (3) the impact of the improvement of control measure, diagnosis and treatment techniques on preventing the spread of novel coronavirus. Since the COVID-19 first large-scale outbreak in Wuhan, scientists and medical staff had little knowledge about this newly emerging disease, and there were no effective diagnosis, treatment and control schemes, resulting in high CCOPSPTT and DOPSPTT in Wuhan. However, as the anti-epidemic work have developed, other cities have continuously improved control measures, diagnostic and treatment techniques, to ensure that patients and carriers have been diagnosed and treated timely, thus prevented effectively the spread of the epidemic [32, 33, 37]. As a result, these cities have fewer deaths and confirmed cases of COVID-19 than Wuhan.

It is the most noteworthy that we studied the rise period of COVID-19 when China implementing the strictest control. The impact of city types on confirmed cases and deaths through GDP, population and spatial distance may be the extension of their original impact before the implementation of government’s control measures. More specifically, nearly 5 million people left Wuhan for other cities before its shutdown [12]. After these people arrived at new cities, differences in GDP, population and other aspects among these cities would have led to the variance of virus transmission before the implementation of Chinese government’s epidemic control in a national scale [1]. In addition, there is an incubation period of COVID-19 and some infected persons are asymptomatic. Hence, carriers of the novel coronavirus continued to spread it during the period of government epidemic control. Thus, influence of city types on CCOPSPTT and DOPSPTT via GDP, population and spatial distance would not be fully eliminated by the implementation of control measures. In this case, city sizes had a significant positive correlation with CCOPSPTT and DOPSPTT in our studied periods.

### Influences of socio-economic determinants, climate, spatial distance and time on the confirmed cases and deaths of COVID-19

Our results showed that socio-economic determinants have the positive and greatest contribution to variability of CCOPSPTT among cities outside Hubei province (Fig 1, Table 4). The higher contribution to CCOPSPTT from than that from population (Fig 1) may be due to that GDP reflects people’s income and consumption ability, better affecting population mobility and activity which was more closely related to the spread of the virus [46, 50]. Time and DW were found the second and third contributing factors affecting CCOPSPTT (Fig 1). This was consistent with the basic theory of epidemiology that the confirmed cases would increase continually over time [5, 51] and with the finding that regions closer to the outbreak of COVID-19 are more easily infected [33, 48]. GLMMs results also proved that DW has the negative relationship with the CCOPSPTT (Table 4). Wuhan was the outbreak source of COVID-19 and the economic and cultural center of central China. During the Chinese Spring Festival, a greater number of people working, studying, visiting and traveling in Wuhan would return to their hometown. According to the theories of economic geography, the gather of the surrounding residents to the economic and cultural centers to would decrease with the increase of spatial distance [32, 50]. In other words, the number of people who left Wuhan to other surrounding cities would decrease with spatial distance [30]. This may have explained the significant negative correlation between DW and CCOPSPTT (Table 2). The weak correlation between DLPC and the confirmed cases indicated that there was not a second city showed a large-scale COVID-19 outbreak in China from January to February. These could be due to China’s strong control. After January 24, all coronavirus patients and carriers either from Wuhan and Hubei province or contacted with people from this region were quarantined at home, potentially having prevented epidemics from a second outbreak [32, 36, 37]. In addition, our results showed that relative air humidity had the negative influence on CCOPSPTT, while average temperature and solar radiation intensity displayed positive influences (Table 4). This echoed the findings reported by a few previous studies [8, 10, 27]. Dalziel et al. [50] and Liu et al. [1] proved that slightly higher temperature and solar radiation intensity and lower relative air humidity in low temperature environment were conducive to the survival and transmission of virus. Qing et al. [8] analyzed the data of infectious diseases in the Ming and Qing dynasties (AD 1368–1901) across several hundred years, and concluded that the viral transmission and deaths were related to climate. However, in this study, we used the positive confirmed cases and deaths of COVID-19 in five consecutive days and the average of climate factors in analyzing their relationship. Compared with the data of daily-variety (or daily analysis unit), our treatment reduced the number of duplicate samples, but the use of average of climatic factors may ignore the effect of abrupt changes of climate factors on viral transmission in the short term (5 days); which may have slightly affected the accuracy of our research.

There was a subtle difference in the contributions of socio-economic determinants, spatial distance, climate and time to CCOPSPTT between cities in Hubei and other provinces (Fig 1 and Table 3). This might be determined by the scale effect. As previous studies suggested, climate changed little in a small region, but population mobility and frequent interpersonal communication caused by population and spatial distance would change more so that could affect viral transmission more [10, 51]. Thus, the contributions of socio-economic determinants and DW to CCOPSPTT in Hubei province were higher than that of other provinces (Fig 1). In comparison, all climate factors showed no significant correlation with CCOPSPTT in Hubei province, while an opposite trend was observed in other provinces (Fig 1 and Table 4).

The confirmed cases was the main contributor to the deaths, which accounted 40.61% and 44.06% for the variance of DOPSPTT in Hubei and other provinces, respectively (Fig 1, Table 4). This is consistent with people’s common agreement that the higher the number of confirmed cases, the higher the number of deaths when an epidemic goes on under the absence of an effective vaccine. Conversely, impacts of GDP, population and spatial distance on DOPSPTT varied between Hubei and other provinces (Fig 1, Table 5). This difference may be caused by China’s strong control [36, 37]. According to the epidemic model without government’s control, the death toll would significantly correlated with the confirmed cases, spatial distance, population mobility and time [33, 48]. After government’s control, the impact of these factors on deaths would be reduced. However, the extent of reduced impact of these factors on death varied with different control measures. For example, CCOPSPTT and GDP that were strongly associated with death (accounted for the largest variance of DOPSPTT among different cities), still affected deaths after implementing control measures (Fig 1). Conversely, the effect of other factors that low associated with deaths may have not been changed by the control measures [32, 37]. In the cities of Hubei province, due to the implementation of many strong control measures, DOPSPTT in Hubei province only showed association with CCOPSPTT and GDP.

Outside Hubei province, population, DW and time have significant correlation with DOPSPTT (Table 5). This may be because of the not as strict control measures in these places as that in Hubei province. Although China imposed nationwide limitation on population mobility at the end of January, the extent to which such controls limited the spread of the virus depended on many factors. For example, (i) the level of the local epidemics when control interventions (*e*.*g*., social distancing, lockdown, etc) were implemented; (ii) the promptness (*e*.*g*., whether the lockdown implementation was abrupt or slowly started) and intensity of such interventions measures; and (iii) the extent of further measures such as testing and isolation of asymptomatic cases [14, 22, 31]. On the national scale, these control measures might be different between provinces. The influence of the control measures on the reduced impact of our studied influencing factors on deaths was not as significant as that in Hubei province. In this case, population, DW and time had significant correlation with the deaths (Table 5). In terms of climate-related factors, due to their loose correlation with deaths, government’s control measures may have prevented them from having a significant impact on DOPSPTT in both Hubei and other provinces.

## Conclusions

Our results found that city sizes had a significant impact on the confirmed cases and deaths of COVID-19 over the population size of 100,000 (COPSPTT and DOPSPTT, respectively), but this effect differed between Hubei and other provinces. The results of GLMMs and HPA showed that the confirmed cases and deaths among different city types were affected by socio-economic determinants, climate and spatial distance and time. Socio-economic determinants were the dominating factor affecting COPSPTT among different cities, while the deaths were mainly determined by the confirmed cases and GDP. Socio-economic determinants and time had positive effect on while spatial distance negative correlated to the confirmed cases and death among different cities. In addition, our results showed that relative air humidity had negative influence on CCOPSPTT while average temperature and solar radiation intensity displayed positive influences. The different influences of socio-economic determinants, climate factors and spatial distance on the confirmed cases and deaths between Hubei and other provinces may be due to China’s strong control measures (more strict in Wuhan and Hubei province) that reduced the spread of novel coronavirus. In conclusion, our study indicated that large cities have a higher risk of COVID-19 infection than small cities. Home quarantine and restriction of population mobility are effective ways to control virus transmission. Therefore, minimization of the population mobility can effectively protect people from COVID-19 infection during its rise phase.

## Supporting information

S1 Data (ZIP)
